# Exploring the restorativeness of different hydrodynamic landscapes in world natural heritage sites

**DOI:** 10.3389/frcha.2025.1506392

**Published:** 2025-02-12

**Authors:** Ping Zhang, Tongyao Zhang, Zexuan Chen, Qianyi He, Ke Luo, Jinpeng Li, Yanbin Yang, Qingjie Zhang, Xuan Wang, Limin Han, Mingze Chen, Fupei Zhao, Xiaoqing He, Saixin Cao, Xiaoqing Xu, Guangyu Wang, Xi Li

**Affiliations:** ^1^ College of Landscape Architecture, Sichuan Agricultural University, Chengdu, China; ^2^ School of Architecture and Urban Planning, Chongqing University, Chongqing, China; ^3^ School of International Education, Chengdu Agricultural College, Chengdu, China; ^4^ Faculty of Forestry, Department of Forest and Resources Management, University of British Columbia, Vancouver, BC, Canada; ^5^ Department of Landscape Architecture, School of Architecture and Urban Planning, Tongji University, Shanghai, China

**Keywords:** audiovisual perception, hydrodynamic landscapes, restorative effect, virtual reality, Jiuzhaigou World Natural Heritage Site

## Abstract

Audiovisual environmental perception has been the focus of numerous empirical studies. This study employs virtual reality (VR) to explore how different hydrodynamic waterscapes in Jiuzhaigou World Natural Heritage Site affect physiological and psychological restoration in youth. According to the results, audiovisual interactions, particularly with water sounds and birdsongs, significantly enhance physiological restoration compared to visuals alone. High-intensity hydrodynamic landscapes, regardless of birdsongs, exhibit the highest physiological restoration. There is a linearly positive correlation between physiological restorativeness and hydrodynamic landscapes. Medium-intensity hydrodynamic landscapes with rich forms are most psychologically restorative. In low-medium-intensity settings, visuals contribute more to psychological restoration than soundscapes. It is further found that waterscapes rich in flora and fauna feature a higher level of biodiversity. In the waterscapes with both elements of vegetation and water, the restorativeness of plant and animal resources is greater than that of water. This work highlights the need to focus on the application of different hydrodynamic landscapes in urban areas and the conservation of World Heritage Sites.

## 1 Introduction

Under the significant pressure and high degree of sedentary work in modern life, individuals often have limited time and motivation for outdoor activities. This trend leads to an increased risk of physical and psychological ailments among youth, characterized by a rapidly declining attention span and accumulating mental fatigue. As a pivotal force driving social development and progress, factors that induce restorative effects on youth warrant comprehensive research.

Studies have shown that green spaces play a positive role in improving mood, alleviating work pressure, and preventing and managing mental illness (1). Among natural environments, blue spaces are considered the most favored, exerting a significant impact on enhancing mental health and psychological well-being (2). The broader utilization of blue–green spaces to promote individuals' health and well-being has become a key issue in creating current health-promoting environments (3). However, studies on the attraction of freshwater blue spaces are limited (4).

Loudness and the pressure level of water sounds, the two components of hydrodynamic intensity, are considered indicators for the restorativeness of water sounds. Water sounds, a significant factor in blue–green spaces, have a remarkable influence on the restorativeness of the environment in terms of acoustic loudness (or sound pressure level) (5). Loudness refers to humans' subjective auditory sensation of sounds and can be represented in decibel values (6). The sound pressure level (SPL) of water varies with water velocity, falling height, and impact material, among other factors (7), with changes in water sound source mainly affected by water velocity. Human auditory perception is reported to vary from enjoyment to like to dislike and then to complete aversion (8). Research on the effects of audiovisual interaction in urban environments has revealed that the availability of visual and auditory information significantly influences the perception of auditory and visual elements. When visual and auditory modalities are simultaneously engaged, individuals typically perceive communicative signals more intensively. Compared to a single sense, multisensory inputs can convey deeper and more comprehensive information, satisfying the human brain's need to integrate the external world through different sensory channels (9, 10). Nature-related audiovisual stimuli are considered more beneficial to health compared to visual stimuli alone (11).

With natural habitats shrinking due to urbanization, restorative natural environments have become scarce resources in cities. In response to this, virtual reality (VR) technology is employed to bridge the gap between people and nature by providing simulated audiovisual landscapes. This allows individuals to conveniently experience nature, making virtual natural environments a valuable tool for alleviating various physical and mental ailments (12).

According to the United Nations Educational, Scientific, and Cultural Organization (UNESCO), World Natural Heritage Sites refer to natural areas that are considered to have “outstanding universal value” from an aesthetic or scientific perspective. These sites are recognized for their unique geological and ecological features, biodiversity or natural beauty that is considered important for the whole of humanity. As unspoiled natural environment offering visitors unique and wonderful experiences, World Natural Heritage Sites can relieve modern people from stress, tension and fatigue caused by decreased directed attention, and reduce the incidence of cardiovascular diseases (13). For depressed patients, mental stress level can be maintained at an ideal state in at least one month after returning to normal life (14).

Jiuzhaigou is a World Heritage Site characterized by its rich blue and green spaces. The study of such spaces aligns fully with the restorative environmental experiences of people (15). However, there are few studies that take Natural World Heritage Sites as a research object, most of which focus on environmental perception, place attachment, and visitor loyalty (16–19). These studies on audiovisual interactions predominantly focus on urban environment. Few studies have examined World Heritage Sites from the perspective of restorative effects.

Therefore, this study aims to explore the blue–green spaces of the Jiuzhaigou World Natural Heritage Site using virtual reality (VR) technology, with a focus on understanding the varying physiopsychological restorativeness of the different hydrodynamic waterscapes in Jiuzhaigou for youth. In particular, we examine differences in audiovisual interactions and pure visuals regarding their effects on promoting physiological restorativeness. Furthermore, we consider disparities in physiopsychological restorativeness among youth resulting from exposure to the different hydrodynamic waterscapes in Jiuzhaigou, and determine whether there exists a hydrodynamic landscape with optimal visual psychological restorativeness for youth within the Jiuzhaigou World Natural Heritage Site.

## 2 Methodology

### 2.1 Research area

**Figure 1:**
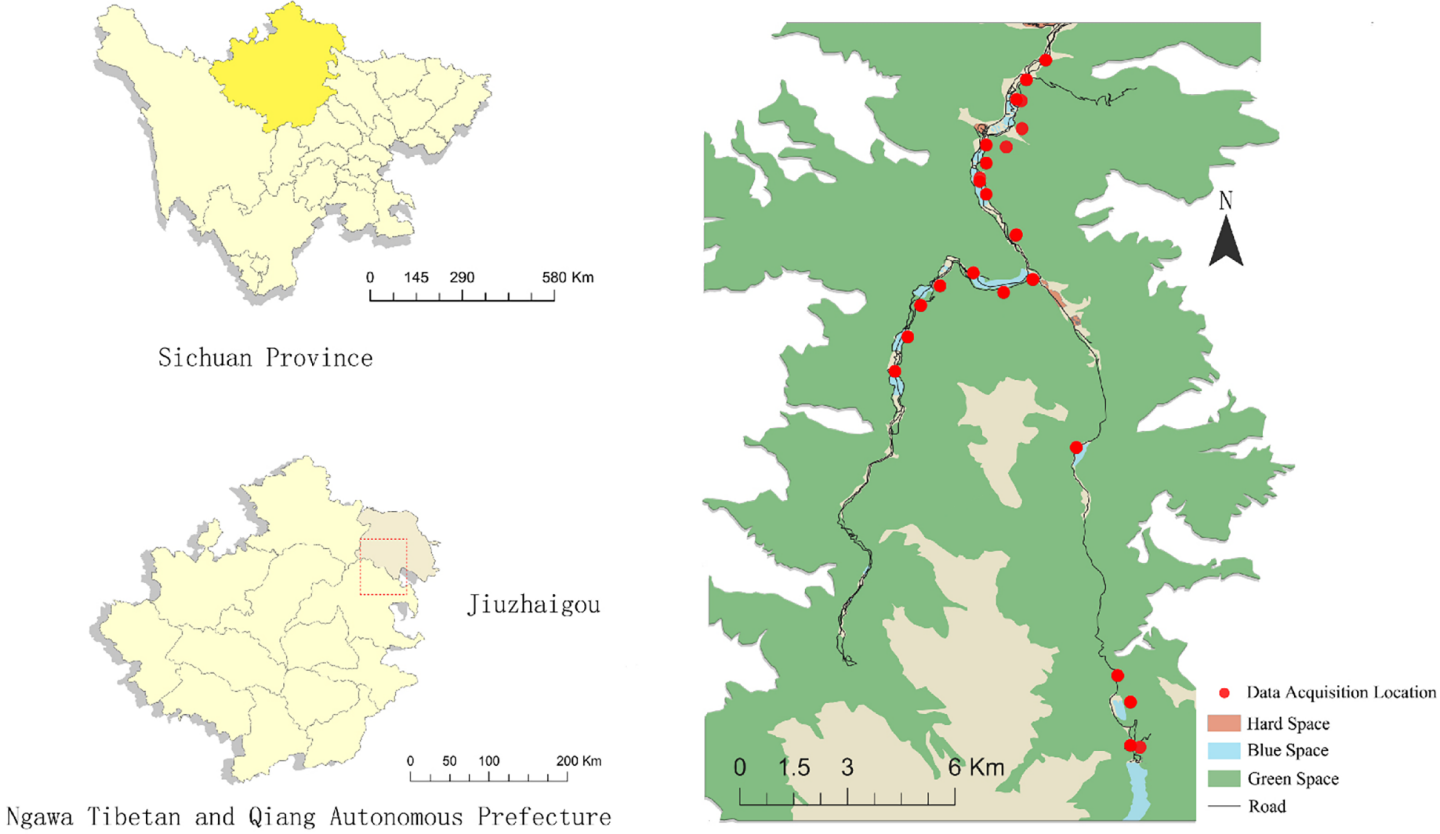
Map of the study area.

Jiuzhaigou is situated in the central and southern parts of Jiuzhaigou County, Sichuan Province, China, covering a total area of about 620 km^2^. Approximately 52% of this area is comprised of virgin forests, boasting rich animal and plant resources, including rare species. This abundance is attributed to the presence of nine Tibetan villages in the valley. Jiuzhaigou Valley, characterized by its plateau traverse, Haizi groups, waterfall clusters, and flowing beaches, stands out as one of China's premier scenic spots for its water features. Springs, waterfalls, rivers, beaches, and 108 Haizi (a term used by locals to refer to the naturally formed, high-altitude lakes found in Jiuzhaigou) form a vibrant jade basin, cherished for its ecological significance, scientific value, aesthetic appeal, and tourism potential (Figure 1).

### 2.2 Research framework

**Figure 2:**
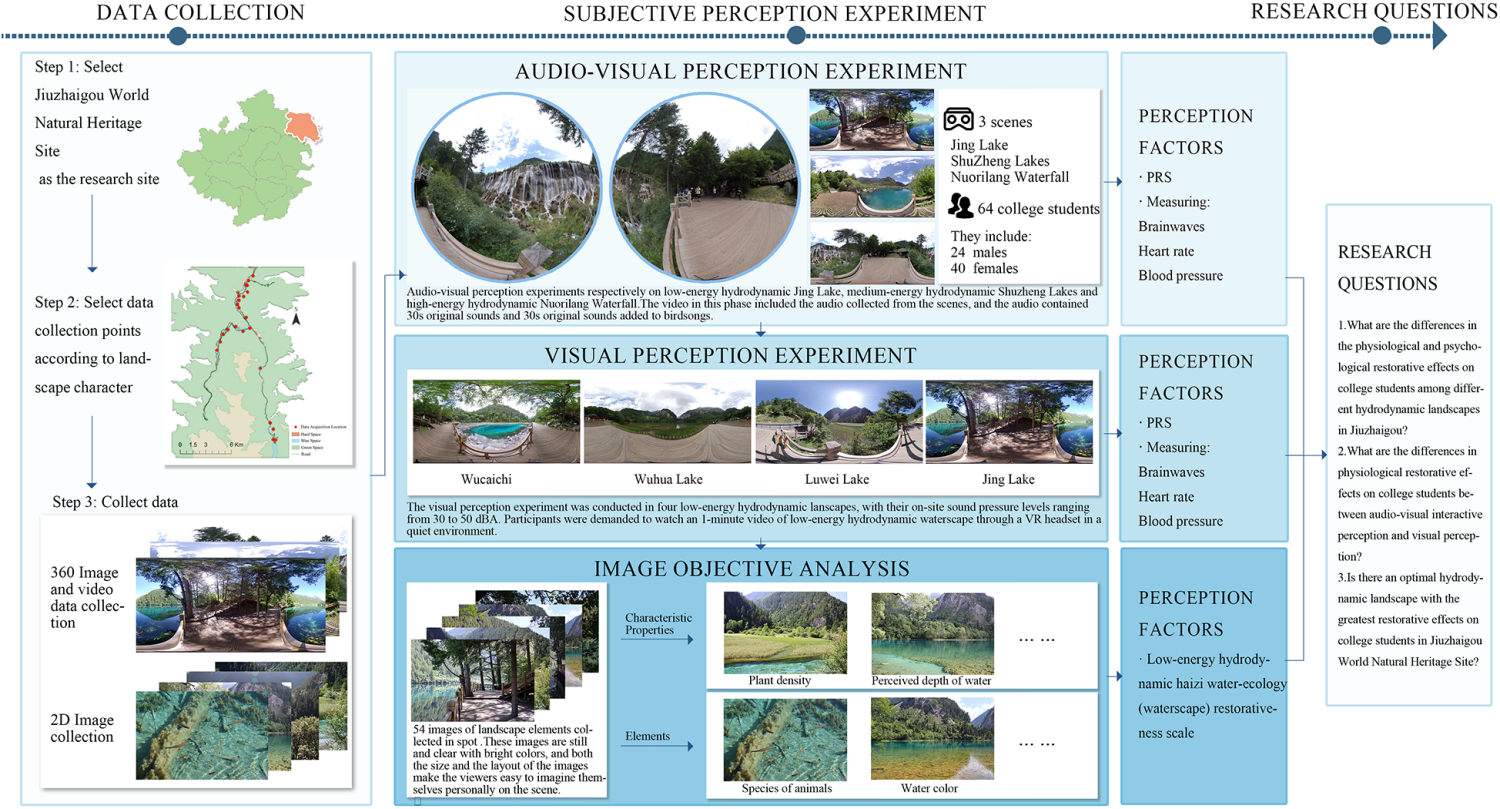
Experimental framework used in this study.

The Jiuzhaigou Scenic Area, located in Aba Tibetan and Qiang Autonomous Prefecture, was chosen as the research site, with 23 image collection points selected according to the landscape environment in Jiuzhaigou for the collection of panoramic video images and two-dimensional water environment factor images. Based on the sound pressure level measured in the field, the collected waterscape points are divided into low, medium, and high hydrodynamic landscapes. Psychological data measurements and scale tests were conducted in the laboratory to explore the influence of different hydrodynamic landscapes on the restorativeness of youth. During the preliminary research process, we observed that low hydrodynamic landscapes (Haizi) feature more abundant waterscape elements, particularly biological habitats, compared to other hydrodynamic shoals and waterfalls. Further exploration was undertaken to investigate the restorative effects of the Haizi components, characteristics, and elements on youth and determine the presence of a hydrodynamic landscape with optimal visual psychological restorativeness for youth within the Jiuzhaigou World Natural Heritage Site (Figure 2).

### 2.3 Collection and processing of experimental data and materials

Before the experiment, panoramic and single-frame images and sounds were collected at 31 points located at lakes, waterfalls, and beaches in Shuzhenggou (Shuzheng Valley), Rizegou (Rize Valley), and Zechawagou (Zechawa Valley) from 26 to 31 July 2022. An Insta 360 (15–20 megapixels and 4/3-in. sensor) and Canon m50 (20–30 megapixels, APS-C sensor, LSO100-25600 standard ISO sensitivity) were employed to collect panoramic and single-frame images, respectively. Considering color, shooting climate, resolution, and representativeness, the panoramic videos and images were collected without people to ensure that the subject focused on the landscape (20). Images and videos were collected during 9–11 a.m. and 2–4 p.m. to ensure similar weather conditions (in July 2022, the average daytime temperature in Jiuzhaigou for the whole month was 30°C, with 85% relative humidity). We selected the shooting locations based on the suggestions of Jiuzhaigou staff, tourists, and residents. For the acquisition of the 360° panoramic videos and images, the Insta 360 panoramic camera was placed on a tripod with the lens at the same height as the human eye (1.2 m). A ZoomH5 recorder, outdoor directional microphone (RODE NTG4), and sound level meter (Aihua 6228) were used to collect natural sound data of water sounds and bird songs.

The minimum sound pressure that can be perceived by individuals with normal hearing is 0 dB (1,000 Hz). However, in general, sound with a stable sound pressure level within 10–15 dB is difficult to perceive. Therefore, this study defines low and medium hydrodynamic intensity underwater sound within the ranges of 30–50 dBA and 51–70 dBA, with high hydrodynamic intensity sounds exceeding 71 dBA. Haizi in Jiuzhaigou are classified according to the hydrodynamic landscape (Supplementary Map). By combining field investigation observations, network comparison, and test requirements, we selected four Haizi landscapes as representatives of low hydrodynamic landscapes, and Shuzheng Lakes and Nuorilang Waterfall as representatives of medium and high hydrodynamic landscapes, respectively. Water panoramic videos of these six landscapes with different hydrodynamics were employed as the test materials (Table 1).

**Table 1:** Description of the study sites.

Hydrodynamic landscape	Sound pressure level	Example	Meter test of original sound level	Landscape features	Image	Panoramic VR video
Low hydrodynamic andscape (Haizi)	30–50 dBA	Wucaichi	32.7 dBA ± 3.4	The scale of the water body is small, the color changes are rich, and the waterfront is composed of rocks.	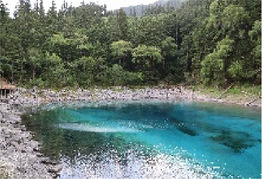	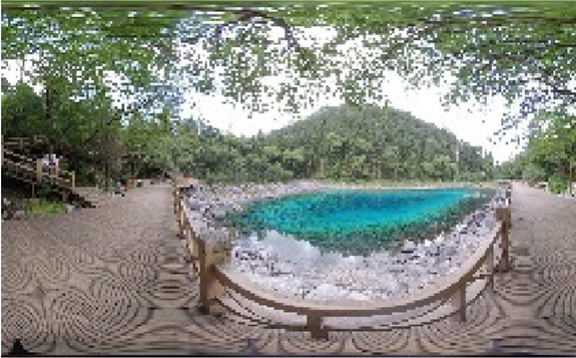
		Wuhua Lake	36.9 dBA ± 3.8	The water body has high transparency, rich color changes, and abundant moist plants.	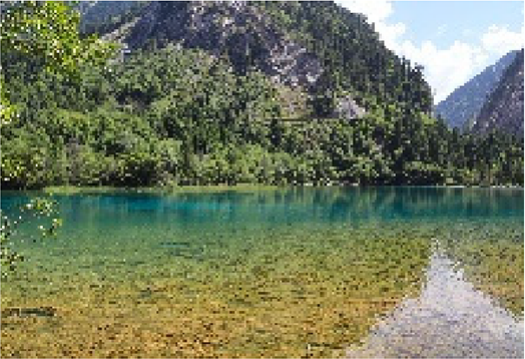	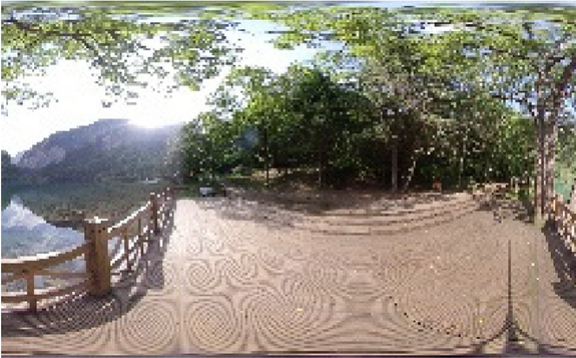
		Luwei Lake	47.4 dBA ± 3.8	Typical banded water body with high surrounding openness and abundant moist plants.	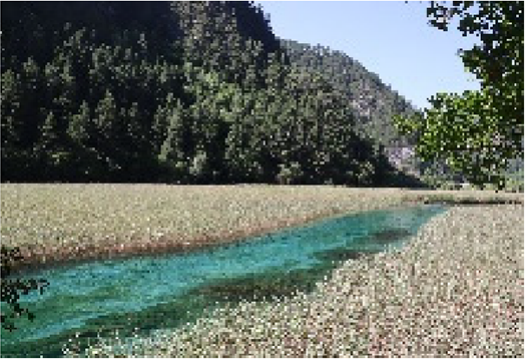	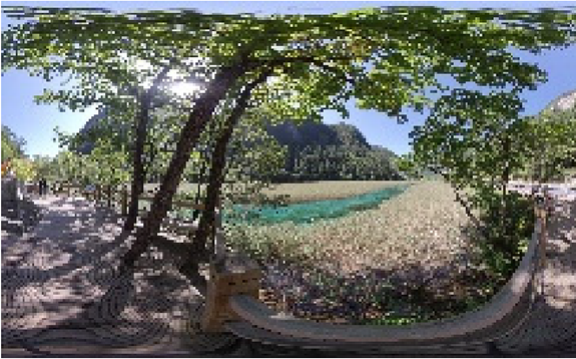
		Mirror Lake	32.7 dBA ± 3.4	The perception depth of the water body is deep, the reflection types in the water are rich, the reflection of complete clouds, mountains, and plants can be seen, and the environment is rich in bird songs.	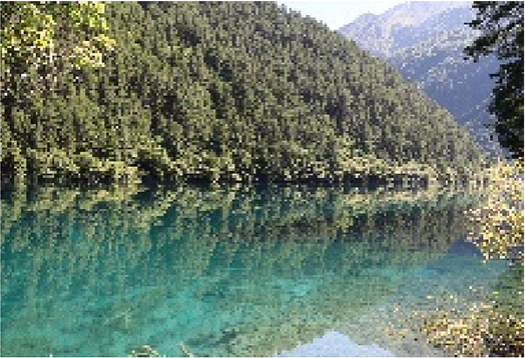	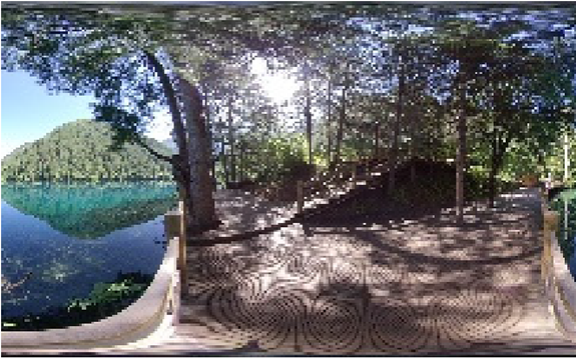
Medium hydrodynamic landscape (combined landscape)	51–70 dBA	Shuzheng Lakes	56.2 dBA ± 0.8	The plant layer is rich; the water body is mainly blue; the water sound is clear.	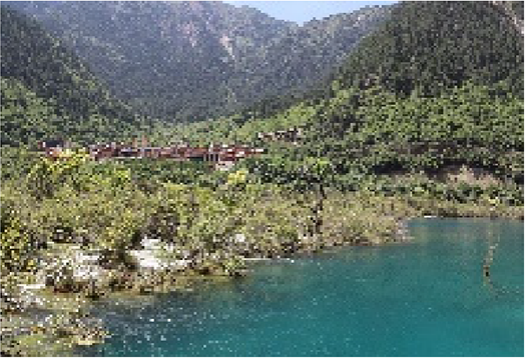	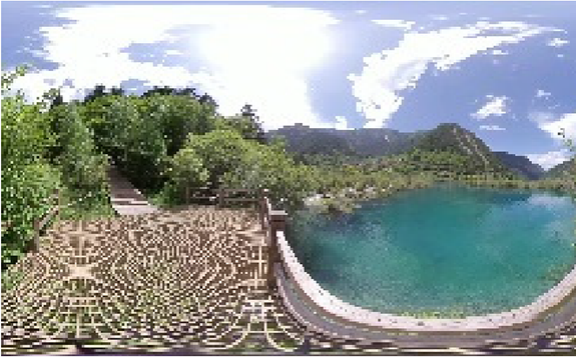
High hydrodynamic landscape (waterfall)	≥71 dBA	Nuorilang Waterfall	71.1 dBA ± 0.6	The plant level is rich, the water quantity is large, the flow rate is large, and the water sound is loud	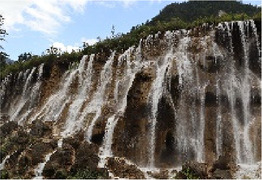	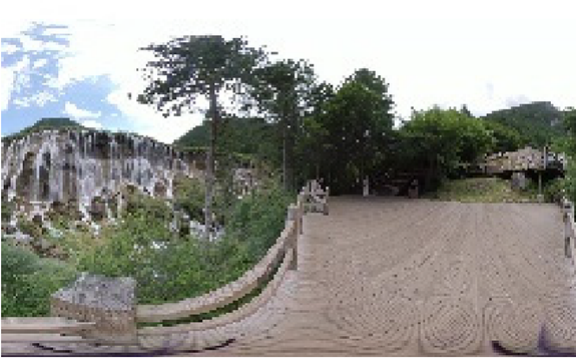

**Figure 3:**
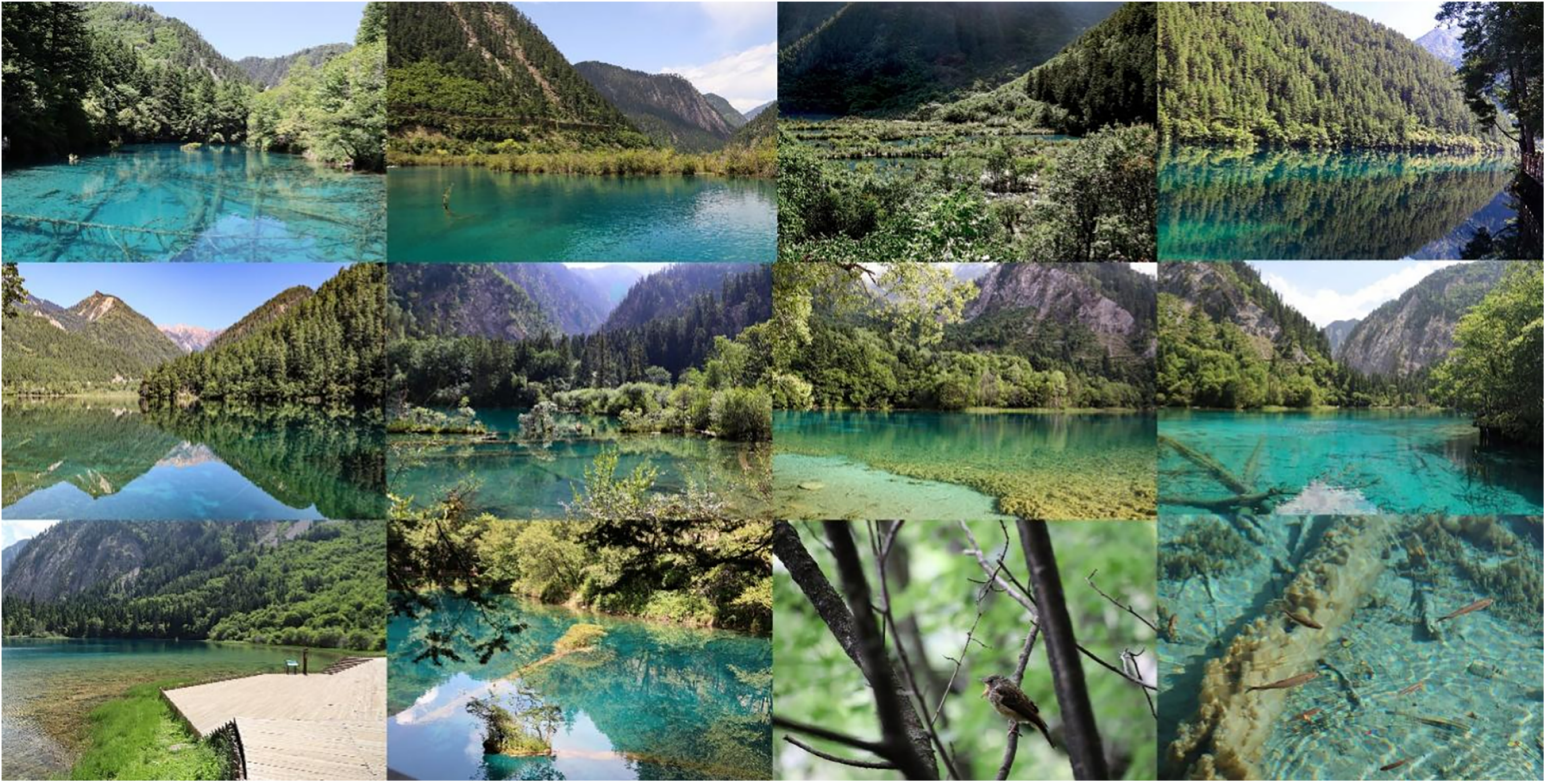
Examples of the 54 single-frame photographs for the perception restorativeness scale of the low hydrodynamic landscapes (Haizi).

We collected 31 panoramic images, 56 soundscape data, and 1,086 single-frame images at 31 points. Among the single-frame images, we retained 100 after three rounds of screening (select images with high clarity, clear composition, and no obvious distracting elements). Following this, 15 graduate students and 10 teachers of landscape architecture determined 54 single-frame photographs as test materials according to the test requirements and subsequently conducted the necessary exposure processing based on maximizing the fidelity (Figure 3). Note that single-frame photographs depict materials containing water bodies, plants at water edges, and animals such as fish and birds. For the collected soundscape materials, the members of the research team compared the external sounds with the embedded sounds, and selected the bird song soundscape in Wucaichi Forest with the most abundant and prosperous bird song ecological niche as the test materials. Adobe Audition 2022 was then used to complete the noise reduction and cleaning process. To ensure that the measured value (dBA value) was as close as possible to the participants' subjective feelings, we introduced A-rate weighting.

### 2.4 Participant**s**

A total of 64 Chinese youth (24 males and 40 females) were selected to participate in the experiment. Among them, 34 were university students and 30 were graduates (24 undergraduates and 10 postgraduates). All participants satisfied the following criteria: (1) they were aged from 19 to 26; (2) their majors covered the fields of landscape architecture, finance, environmental engineering, animal science, medicine, ecology and economics. Due to the nature of their professions, they all needed to spend extended time conducting experiments in sealed laboratories, engaging in physically demanding research of natural or social science, or spending hours engineering drawing in the office. As a result, they experienced high levels of learning pressure and both physical and mental fatigue.; (3) they generally had consistent vision, hearing, and color recognition abilities, good physical and mental health levels, and no history of drug abuse, alcoholism, or smoking; and (4) they visited green spaces one to three times per week, with the green spaces typically on campus or close to schools and homes (e.g., nearby parks). The study was conducted in accordance with the Declaration of Helsinki and was approved by the Human Research Ethics Committee of Sichuan Agricultural University.

### 2.5 Assessment procedures

We measured the differences in the physiological and psychological restorativeness of different hydrodynamic landscapes using physiological and psychological assessments and a questionnaire survey. The physiological assessment data was collected by recording the heart rate (HR), blood pressure (BP), and electroencephalogram (EEG). The psychological assessment data was collected by applying the perception restorativeness scale (PRS). The questionnaire survey was conducted using a PRS specifically designed for low hydrodynamic landscapes (Haizi) by the study group.

#### 2.5.1 Physiological data measurements

The blood pressure [systolic (mmHg), diastolic (mmHg)] and heart rate of the participants were measured on the left arm using an electronic sphygmomanometer (OMRON HEM-7135) to indicate the relaxation degree of the body. The HR is considered a physiological response indicator to physiological stimuli and psychological stress (21). EEG is an oscillogram of electrophysiological signals that reflects the neurophysiological activities of brain neurons on the cerebral cortex or the scalp (22). It contains a large amount of information about brain activity that is closely related to human thinking, physical activity, mental state, and physical and mental health (23). Different wave bands can reflect different emotional states and cognitive abilities (24). The brain signals were measured in this study with a Wise Brain Instrument (Sichiray 03) at three waves (theta, alpha, and beta). Theta (*θ*) waves range from approximately 4–8 Hz and are typically associated with deep relaxation, meditation, and mild sleep stages such as (rapid eye movement (REM) sleep. They are often considered as the brainwaves related to creativity and subconscious thinking. At this frequency, the abilities of memory, learning, and emotional integration may be enhanced as the brain enters a state of marginal consciousness, which is conducive to the formation of new ideas and concepts. Alpha (α) waves, which range from approximately 8–12 Hz, indicate relaxation in a state of consciousness and can often get intensified while sitting down with the eyes closed. They are associated with feelings of relaxation, tranquility, and a balance of physical and mental states. Enhanced alpha wave activity suggests that an individual may be in a state of idle thinking or enhanced creativity. Alpha wave activity often increases when individuals are in contact with nature, performing deep breathing exercises, or engaging in relaxation activities. Beta (β) waves range from approximately 12–30 Hz and are associated with daily alertness, concentration, decision-making, and problem-solving activities. High-frequency beta waves are related to states of anxiety, stress, or panic. However, moderate beta wave activity is healthy, indicating that the brain is in an alert and active state, capable of effective logical thinking and critical analysis.

#### 2.5.2 Psychological data measurements

Scales are typically used to measure psychological restorativeness, with PRS as the most representative (Appendix A). Developed by Hartig et al. (25), the PRS contains four items and 26 projects (20). Due to the cultural differences and different language habits in the semantics of different countries, Wang (26) applied the Chinese version of PRS to the restorativeness assessment of urban park landscapes, validating the reliability of the Chinese version of PRS (27, 28). The PRS used for the psychological measurements in this study is based on the modified version of Huang et al. (29), consisting of 18 items in four dimensions (distance, extension, charisma, and compatibility), and modified according to the attention restorative theory by Kaplan et al. (27) (Appendix A).

#### 2.5.3 Questionnaire survey

This study further developed the PRS for low hydrodynamic landscapes (Haizi). Based on field investigations and literature reviews, the scale was ultimately determined to consist of three dimensions: the scale for the restorativeness of Haizi components; the characteristics of Haizi (Appendix B); and the elements of Haizi (Appendix C). The Haizi characteristics that vary continuously can generally be described by an interval, while the Haizi elements have discrete features. Each level is rated on a perception restorativeness scale from 1 to 7, where 7 represents the highest positive evaluation (considered the most restorative), and 1 represents the lowest evaluation (considered the least restorative).

### 2.6 Experimental procedures

The experiment was performed from October 15 to November 17, 2023, during 7–8:30 p.m. each day. The laboratory maintained a consistent physical environment, namely, it was quiet (noise below 25 dB), clean (interior space of 10 m^2^ containing a set of desks and chairs and no windows), with appropriate light, temperature (20°C–23°C) and relative humidity (about 45%–55%), and without disturbances. The VR device (HTC VIVE Professional Version) was set up and debugged by staff in the laboratory before measurements. The device contained two positioners and a headset, with the following parameters: 90 Hz refresh rate; 110° maximum field angle; 2,880 × 1,600 resolution; smooth and clear video-broadcasting; built-in high-resolution-certified headphones; and good audio sound quality. When watching the videos, the participants could freely turn their heads to simulate the real walking experience. The test lasted for approximately 45 min.

**Figure 4:**
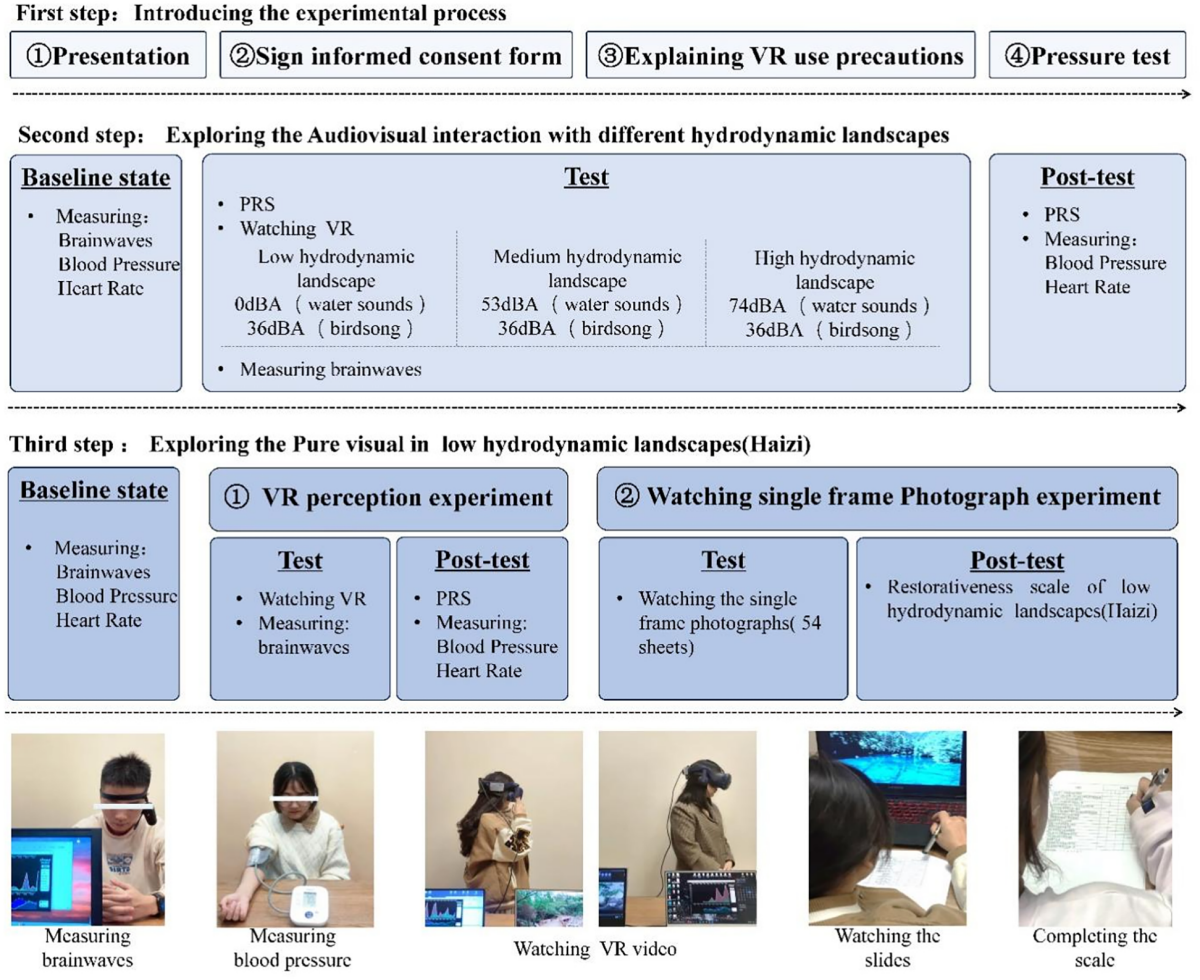
Experimental procedure experienced by the participants.

Upon arriving at the laboratory, the participants were involved in three steps for the test procedure. The first step involved informing the participants about the test's purpose, procedure, and any necessary precautions by the staff. Participants filled out an informed consent form, which included consent for human image use. After the first step has been completed, participants then independently completed 1-min mental arithmetic tasks. For the second step, the blood pressure monitor was employed to test the participants’ blood pressure and heart rate, and an EEG then recorded brainwave data for 1 min. Participants sequentially watched 1-min panoramic videos featuring landscapes with low, medium, and high hydrodynamic intensities (Mirror Lake, Shuzheng Lakes, and Nuorilang Waterfall) in quiet surroundings through VR headsets. The video comprises 30 s of original sounds and 30 s of original sounds with bird songs. During this process, staff members monitored and recorded the participants' brainwave data in real-time. After viewing each hydrodynamic landscape through VR, participants removed the VR devices and completed a PRS. The VR devices were then worn again for the next test. Following this, participants took a 6-min rest to ensure that they experienced the same level of fatigue before the next stage of the test. For the third step, the participants had their blood pressure and heart rate monitored again using the blood pressure monitor, followed by the recording of brainwave data using the EEG for 1 min. Participants then sequentially watched a 1-min visual panoramic video featuring low hydrodynamic landscapes (Mirror Lake, Wuhua Lake, Luwei Lake, and Wucaichi) in quiet surroundings through VR headsets. Staff members monitored and recorded the participants' brainwave data in real-time during this process. After watching the video, the blood pressure and heart rate of the participants were measured, and they completed the PRS. The staff collected the completed scales, and participants were allowed a 2-min rest. Following this, participants viewed single-frame photographs presented in slides. The slide show began with a 60-s blank and then displayed 54 still, clear, and brightly colored images of landscapes. The size and layout of the photographs aimed to immerse the participants into the scene. During the viewing of the images, participants could freely navigate the slides. Finally, the participants completed the 15-min Haizi perception restorativeness scale (PRS) (Figure 4).

### 2.7 Statistical analysis

Statistical analysis was performed with SPSS 26.0 (IBM Corp.). Paired *t*-tests, repeated measures analysis of variance test (ANOVA), and the mean difference were applied to calculate the physiological and psychological state indicators. If large differences were observed among the test results, the least-significant difference (LSD) test was performed. In this study, a *P*-value less than 0.05 indicates statistical significance. A stepwise linear regression model was applied to test the perceptual restorativeness of the constituents, characteristics, and elements of Haizi. A VIF of multiple linear regression values exceeding 10 indicates collinearity among the independent variables and as a consequence, the unreliability of the generated equation model. The model fit is considered acceptable for goodness-of-fit values of the multiple regression greater than 30%.

## 3 Results

In this study, the maximum VIF value of the model is determined as 2.542, indicating that there is no collinearity and that the model is reliable. All dimensions of the model exhibited goodness-of-fit (*R*²) values exceeding 30%, with many exceeding 50%, and the highest reaching 81.5%. Therefore, the model fit is considered acceptable. The results of this study on physiological and psychological data are as follows:

### 3.1 Physiological data

#### 3.1.1 Blood pressure and heart rate data

**Figure 5:**
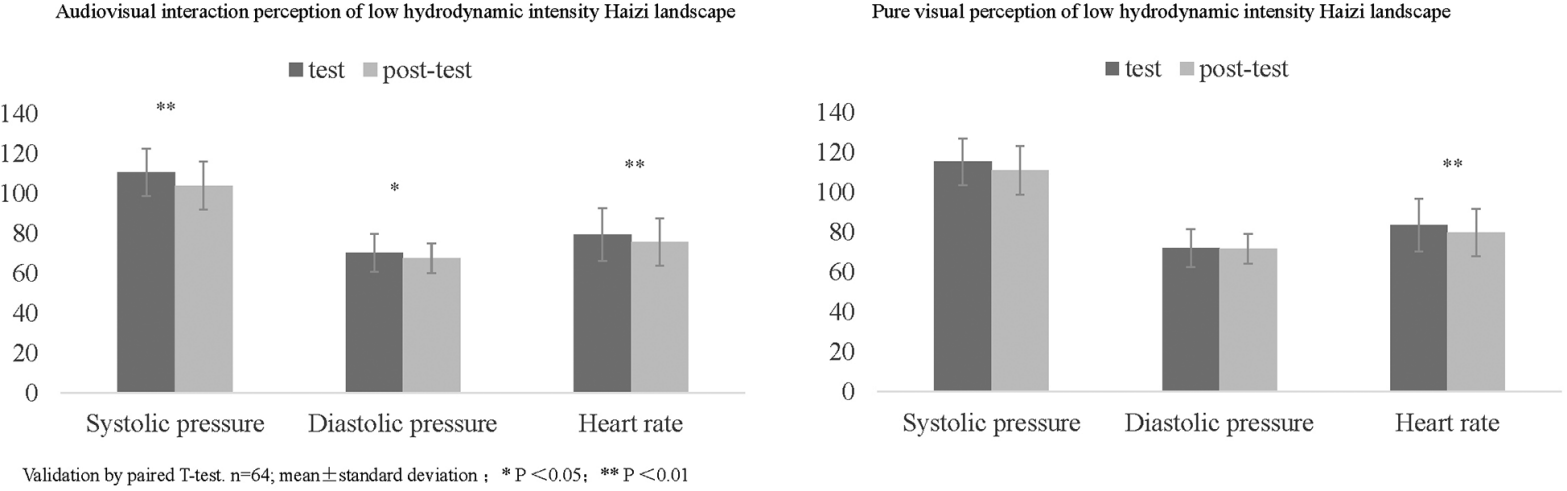
Blood pressure and heart rate data.

Following the audiovisual interaction test of the low hydrodynamic landscape (Haizi), participants exhibit a significant decrease in heart rate (79.22 ± 13.30–75.59 ± 10.78, *P* < 0.01), systolic pressure (110.42 ± 9.22–103.81 ± 8.55, *P* < 0.01), and diastolic pressure (70.16 ± 9.47–67.52 ± 7.22, *P* < 0.05) (Figure 4). Following the pure visual test of Haizi, participants present a significant reduction in heart rate (84.50 ± 14.30–79.37 ± 12.03, *P* < 0.01), while systolic and diastolic blood pressure exhibit minimal changes (Figure 5).

#### 3.1.2 Brainwave data

After completing the audiovisual interaction of different hydrodynamic landscapes, the mean theta, alpha, and beta wave values are observed to increase greatly. Paired *T*-test results show marked differences (*P* < 0.05) between baseline and experimental theta, alpha, and beta wave data for the different hydrodynamic landscape tests. Moreover, the mean values of the three waves peak during the high hydrodynamic landscape (waterfall) test. However, no significant differences are observed in the corresponding ANOVA results (Table 2).

**Table 2:** Brainwave response to the audiovisual interaction of different hydrodynamic landscapes.

Brainwave	Baseline value	Low hydrodynamic landscape (Haizi)	Medium hydrodynamic landscape (combined landscape)	High hydrodynamic landscape (waterfall)	*P*- value	Difference value	*T*-value
Theta wave	156,429.26	232,712.00	242,948.11	262,758.90	0.001** 0.000*** 0.000***	76,282.74 86,515.85 106,329.64	−3.715 −4.672 −5.060
Alpha wave	66,721.13	101,320.11	109,653.13	116,431.77	0.002** 0.000*** 0.000***	34,598.98 42,932.00 49,710.64	−3.340 −4.379 −4.295
Beta wave	45,957.34	62,915.66	68,214.69	73,131.74	0.015** 0.003** 0.002**	16,958.32 22,257.35 27,174.40	−2.575 −3.281 −3.350

*P < 0.05, **P < 0.01, ***P < 0.001.

After the audiovisual interaction test (first 30-s video) of the different hydrodynamic landscapes, the mean values of the theta, alpha, and beta waves are observed to increase greatly. The three paired *t*-test results exhibit significant differences (*P* < 0.05) between the baseline data and the experimental theta, alpha, and beta wave values for the three landscape tests. Moreover, the mean values of the three waves peak during the high hydrodynamic landscape (waterfall) test. However, no significant differences are observed in the corresponding ANOVA results (Table 3).

**Table 3:** Inter-group comparison of brain waves for the first 30 s of the audiovisual interaction of different hydrodynamic landscapes (water sound + no bird songs).

Brainwave	Baseline value	Low hydrodynamic landscape (Haizi)	Medium hydrodynamic landscape (combined landscape)	High hydrodynamic landscape (waterfall)	*P*-value	Difference value
Theta wave	147,320.65	230,030.91	231,558.95	239,302.67	0.001** 0.000*** 0.000***	82,710.26 84,238.30 91,982.02
Alpha wave	66,314.41	108,476.33	106,088.06	108,970.82	0.004** 0.000*** 0.000***	42,161.92 39,773.65 42,656.41
Beta wave	46,391.11	67,237.53	67,611.53	67,863.10	0.035* 0.014* 0.017*	20,846.42 21,220.42 21,471.99

*P < 0.05, **P < 0.01, ***P < 0.001.

The theta waves are observed to increase greatly for the second part of the audiovisual interaction test (second 30-s video) of the different hydrodynamic landscapes. The paired *t*-test results indicate significant differences (*P* < 0.05) between the baseline and experimental theta and alpha values for the three landscape tests, as well as between baseline and experimental beta wave values for the waterfall and combined landscape test. No significant differences are observed for Haizi. The mean values of the three waves also peak during the high hydrodynamic landscape (waterfall) test. Furthermore, there are no significant differences in the theta, alpha, and beta wave values for the three landscape tests (Table 4).

**Table 4:** Inter-group comparison of brain waves after 30 s of the audiovisual interaction of different hydrodynamic landscapes (water sounds + bird songs).

Brainwave	Baseline value	Low hydrodynamic landscape (Haizi)	Medium hydrodynamic landscape (combined landscape)	High hydrodynamic landscape (waterfall)	*P*-value	Difference value
Theta wave	165,537.88	235,393.09	254,337.27	286,215.12	0.006** 0.000*** 0.000***	69,855.21 88,799.39 120,677.24
Alpha wave	67,127.84	94,163.88	113,218.19	123,892.73	0.011* 0.001** 0.000***	27,036.04 46,090.35 56,764.89
Beta wave	45,523.56	58,593.78	68,817.85	78,400.39	0.093 0.004** 0.001	13,070.22 23,294.29 32,876.83

*P < 0.05, **P < 0.01, ***P < 0.001.

Comparing the mean values of the theta, alpha, and beta waves between the first and second 30-s tests indicate that the values of the three waves in the Haizi test decrease after the addition of the bird songs, and increase in the waterfall and combined landscape tests (Tables 3, 4).

In the tests focusing solely on the visual perception of Haizi, significant increases are observed in the mean values of the theta, alpha, and beta waves across the four Haizi landscapes (Mirror Lake, Wucaichi, Luwei Lake, and Wuhua Lake). Wucaichi exhibits the highest theta wave value, while the alpha and beta waves peak for Luwei Lake and Jing Hai, respectively. Paired *t*-test results reveal notable differences (*P* < 0.05) between the data in the baseline and experimental states for the three waves across the four Haizi landscapes. In contrast, the ANOVA results indicate no significant differences in theta, alpha, and beta waves among the four Haizi landscapes (Table 5).

**Table 5:** Brainwave responses to the pure visuals of haizi.

Brainwave	Baseline value	Mirror Lake	Wucaichi	Luwei Lake	Wuhua Lake	*P*-value	*T*-value
Theta wave	127,432.8	235,020.6	242,362.22	235,035.89	230,796.22	0.000***/0.001**/ 0.006**/0.003**	−3.996/−3.790/ −2.978/−3.191
Alpha wave	54,443.33	103,737.03	102,145.62	105,107.84	101,667.2	0.000***/0.001**/ 0.003**/0.004**	−4.256/−3.570/ −3.173/−3.084
Beta wave	34,072.56	65,287.99	62,511.67	63,136.4	63,609.38	0.005**/0.014*/ 0.008**/0.019*	−3.042/−2.591/ −2.849/−2.481

*P < 0.05, **P < 0.01, ***P < 0.001.

According to the physiological data, all low hydrodynamic landscapes exhibit significant physiological restorative effects under pure visual conditions, with no significant differences observed in physiological restorativeness among the four Haizi landscapes. The highest values of theta, alpha, and beta brain waves are found in different Haizi. This may be attributed to both the similarities and partial differences in the features and elements of the waterscape environment of Haizi.

### 3.2 Subjective recovery data

**Figure 6:**
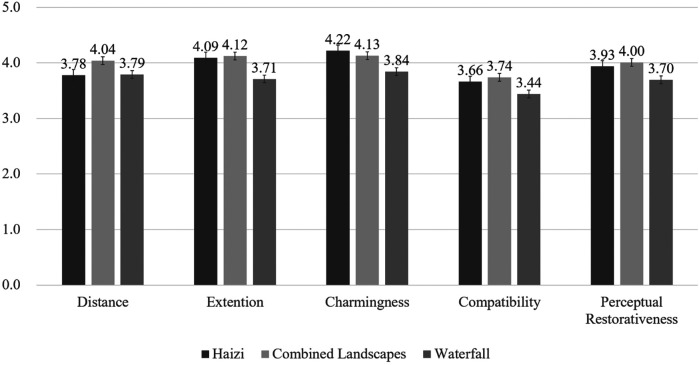
Audiovisual interaction of PRS in different hydrodynamic landscapes. Validation was performed by paired *T*-tests. Data is expressed as mean ± standard deviation. *n* = 64; *P < 0.05; **P < 0.01.

According to the mean test value and standard deviation of the PRS, the different hydrodynamic landscapes can be ranked in terms of their restorative effects as follows (in descending order): combined landscape (Shuzheng Lakes), Haizi (Mirror Lake), and waterfall landscape (Nuorilang Waterfall). The combined landscape (Shuzheng Lakes) ranks number 1 in terms of Distance, Compatibility, and Extension. The top-ranking landscape in Charmingness is Haizi (Mirror Lake).The Haizi landscapes are ranked in terms of their restorative effects as follows (in descending order): Charmingness, Extension, Distance, and Compatibility (Figure 6).

Figure 6 indicates that the Charmingness of the pure visuals of Haizi is significantly higher than that of distance and extensibility. Under audiovisual interaction, Haizi emerges as the most attractive landscape, with its overall psychological restorativeness ranking second, surpassing even that of the waterfall. Based on these findings, low hydrodynamic landscapes (Haizi) exhibit greater restorativeness and more abundant spatial elements, particularly biological habitats, compared to medium and high hydrodynamic landscapes. For low hydrodynamic landscapes, intense visual stimulation may bring about far greater restorativeness than sounds in the environment. Consequently, the low hydrodynamic Haizi landscapes, characterized by minimal disturbance of auditory stimulation, are selected to analyze the restorativeness of the landscape components, characteristics, and elements under pure visuals. This exploration aims to determine whether there exists a hydrodynamic landscape with optimal visual psychological restorativeness for youth within the Jiuzhaigou World Natural Heritage Site.

### 3.3 Multivariate linear regression analysis of the perceived restorativeness scale (PRS) of low hydrodynamic landscapes (Haizi)

#### 3.3.1 Overall restorativeness of the components of Haizi

Table 6 reveals that all values of the four components are positive, with the plant and animal characteristics exhibiting the highest value (*B* = 0.362, *P* < 0.05). The overall restorativeness of Haizi can be calculated as −0.044 + 0.271 × [water characteristics] + 0.362 × [plant and animal characteristics] + 0.213 × [water environment] + 0.202 × [plant and animal environment]. The components of Haizi that contribute positively to overall restorativeness include animal and plant characteristics, water characteristics, water environments, and animal and plant environments, with animal and plant characteristics having the most significant influence on restorativeness. The other three components have similar effects (Table 6).

**Table 6:** Multiple linear regression analysis of the overall restorativeness regarding components of Haizi.

Model	Non-standard coefficient		Standard coefficient	*T*-value	*P*-value	95.0% confidence interval for beta		Collinear statistics		*R*²	Adjusted *R*²
	B	Standard error	Beta			Minimum value	Maximum value	Tolerance	VIF		
(Constant)	−.044	.758		−.059	.954	−1.610	1.521			.776	.738
Water characteristics	.271	.133	.233	2.048	.052	−.002	.545	.724	1.382		
Plant and animal characteristics	.362	.101	.479	3.571	.002**	.153	.571	.520	1.924		
Water environments	.213	.112	.214	1.911	.068	−.017	.444	.745	1.341		
Plant and animal environments	.202	.103	.272	1.949	.063	−.012	.415	.480	2.082		
Implicit variable: overall restorativeness of the Haizi components											

*P < 0.05, **P < 0.01, ***P < 0.001.

#### 3.3.2 Restorativeness of water characteristics

Appendix Table 7 shows that all the water characteristic non-standard coefficient values are positive, with perceived depth exhibiting the highest value (*B* = 0.240, *P* < 0.05). The restorativeness of water characteristics can be calculated as 1.978 + 0.046 × [clearness] + 0.240 × [perceived depth] + 0.178 × [openness] + 0.084 × [surface complexity] + 0.144 × [color richness]. The characteristics that contribute to restorativeness include water transparency, perceived depth, openness, surface complexity, and color richness, with perceived depth having the greatest effect (Appendix Table 7).

#### 3.3.3 Restorativeness of plant and animal characteristics

Appendix Table 8 indicates the plant color richness value to be negative (*B* = −0.163, *P* > 0.05), while the other values are positive. Plant density and plant level richness exhibit the highest values (*B* = 0.419, 0.416, *P* < 0.05). The restorativeness of the plant and animal characteristics can be calculated as 0.934 + 0.419 × [plant density + 0.416] × [plant level richness] + −0.163 × [plant color richness] + 0.091 × plant form richness. Plant form richness, plant density, and plant level richness exhibit positive restorativeness, with the latter two exerting the greatest effects, while plant color richness has a negative restorative effect (Appendix Table 8).

#### 3.3.4 Restorativeness of water environments

Appendix Table 9 indicates that watercolor, waterfront elements, water shape, and reflection have positive effects on restorativeness, with watercolor and reflection exerting the strongest influence. In contrast, the component individual scene has a negative effect (Appendix Table 9). The restorativeness of the water environment can be calculated as 0.739 + 0.348 × [water color] + 0.347 × [waterfront elements] + 0.052 × [water shape] + 0.373 × [water reflection] + −0.219 × [individual scene].

Further analysis was conducted on the components of water reflection, watercolor, and individual scenes. In terms of the water reflection components, Appendix Table 10 shows that clouds have the most significant effect on restorativeness (*B* = 0.435, *P* < 0.05), while plants on the water have a negative effect (Beta = −0.023, *P* > 0.05). The restorativeness of water reflection can be calculated as 1.101 + −0.023 × [plants on the water] + 0.219 × [waterfront plants] + 0.453 × [clouds + 0.201] × mountains (Appendix Table 10). The watercolor components are observed to have a positive effect, with green having the greatest effect (*B* = 0.306, *P* < 0.05) (Appendix Table 11). The restorativeness of watercolor can be determined as 2.238 + 0.222 × [mainly blue] + 0.306 × [mainly green] + 0.154 × [mainly yellow]. Among the individual scene components, plank roads over water exert the greatest negative effect (*B* = 0.381, *P* < 0.05) (Appendix Table 12). Note that the values in Appendix Table 12 indicate negative impacts of the individual scene components, and the higher the value, the greater the negative impact. The negative impact of the individual scene components is calculated as1.369 + 0.236 × [plank roads by the shore] + 0.381 × [plank roads over water] + 0.065 × [viewing platform with guardrails] + 0.051× [fallen trees in the water].

Analysis of the restorativeness of the plant and animal environments shows that both animal and plant species exert positive (and similar) effects (*B* = 0.547, 0.543, *P* < 0.05). The restorativeness of plant and animal environments can be calculated as −0.674 + 0.543 × [plant species] + 0.547 × [animal species] (Appendix Table 13). Through further analysis, it is revealed that submerged plants, emergent plants, wetland shrubs, and wetland trees have significant positive effects, with wetland shrubs exerting the strongest influence (*B* = 0.373, *P* < 0.05). The restorativeness of plant species can be calculated as 0.435 + 0.044 × [submerged plants] + 0.212 × [emergent plants] + 0.373 × [wetland shrubs] + 0.352 × [wetland trees] (Appendix Table 14). Terrestrial and fish are observed to have remarkable effects on restorativeness, with terrestrial demonstrating the most significant influence (*B* = 0.455, *P* < 0.05). The restorativeness of animal species can be calculated as 0.760 + 0.455 × [terrestrial] + 0.433 × [fish] (Appendix Table 15).

## 4 Discussion

### 4.1 Physiological restorativeness of different hydrodynamic landscapes under audiovisual interaction

The above analysis indicates that all the hydrodynamic landscapes exhibit significant restorative effects under audiovisual interaction, regardless of whether there are bird songs or not. However, the differences between groups are not statistically significant. Notably, the highest values of theta, alpha, and beta brain waves consistently appear in waterfalls, indicating that high hydrodynamic landscapes (waterfalls) generally offer the greatest physiological restorativeness. In terms of audiovisually-perceived restorative effects of bird songs, the landscapes rank as follows (in descending order): high hydrodynamic landscape (waterfall), medium hydrodynamic landscape (combined landscape), and low hydrodynamic landscape (Haizi). Moreover, there is a linearly positive correlation between physiological restorativeness and hydrodynamic landscapes. Bird song represents one of nature's restorative sound sources, evoking associations with highly biodiverse environments and vitality (30, 31), enhancing landscape experiences (32), and improving the overall impression and satisfaction with Jiuzhaigou's water environment (33). Hydrodynamic landscapes influence people's perception of bird songs. Higher-level hydrodynamic landscapes do not diminish the perception of bird song, rather they enrich and optimize the spatial ecological niche to some extent, resulting in a certain degree of soundscape richness. Compared to the rapid and orderly sounds of falling water as a background sound, the rich and varied bird songs are highly distinguishable (34). Therefore, when bird songs are added to the continuous water sounds of waterfalls and combined landscapes with high and medium hydrodynamic intensities, listeners are provided with a richer and more pleasant rhythm experience, enhancing their restorative experience in the soundscapes. Pleasant sounds added to a space can greatly enhance acoustic comfort, even if the original sound source is loud (35). Despite the addition of bird songs, the physiological restorativeness of Haizi remains weaker than that of waterfalls and combined landscapes, with its own restorativeness exhibiting a declining trend. Haizi features rich spatial elements, particularly biological habitats, and the reception and processing of visually perceived information are markedly intense. The contribution of bird songs to auditory stimulation is relatively weaker compared to that of visual simulation.

### 4.2 Physiological restorativeness of different hydrodynamic landscapes under pure visuals

According to the physiological brain waves data, distinctions in the restorativeness of various hydrodynamic landscapes persist between audiovisual interaction and purely visual experiences under pure visuals, all low hydrodynamic landscapes exhibit significant physiological restorative effects, with no significant differences observed in the physiological restorativeness among the four Haizi landscapes. The highest theta, alpha, and beta brain wave values vary across different Haizi. This may be attributed to similarities and partial differences in the features and elements of the waterscape environment. Compared to high and medium hydrodynamic landscapes, low hydrodynamic landscapes boast more abundant spatial elements, particularly biological habitats. The contribution of visually stimulating elements to psychological restorativeness may outweigh that of auditory stimulation, with visuals remaining the primary sensory input.

### 4.3 Psychological restorativeness of different hydrodynamic landscapes

According to the data of PRS (Figure 6), we reveal that the Charmingness perceived in the pure visuals of Haizi significantly surpasses that of Distance and Extension. Under audiovisual interaction, Haizi emerges as the most attractive landscape, ranking second in overall psychological restorativeness, surpassing waterfalls. This suggests an inconsistency between physiological and psychological restorative effects, with the latter displaying a nonlinear relationship with hydrodynamic landscapes. When the visual features of waterscapes are prominently displayed, the perception of water sound loudness tends to decrease (36). In addition, Distance, Extension, Compatibility, and the overall perceptual restorativeness ratings of combined landscapes with medium hydrodynamic landscapes rank highest. These landscapes also exhibit the highest psychological restorative effects on youth, with physiological restorativeness following closely behind. This may be attributed to the abundance of visual stimuli, particularly the aesthetic beauty of water flow lines. In high hydrodynamic waterscapes, where visual stimulation is relatively scarce, the influence of water sounds and birdsongs on restorativeness may be decisive. Sounds can convey subjective emotions that visuals cannot express and can compensate for limitations resulting from reduced visuals (37). This aligns with the results presented by Evensen et al. (38), indicating that positive soundscapes can significantly improve psychological states and induce happiness when visual factors are disregarded in perception.

### 4.4 Pure visually-perceived restorativeness of low hydrodynamic landscapes (Haizi)

#### 4.4.1 Restorativeness of the components of low hydrodynamic landscapes (Haizi)

The components of low hydrodynamic landscapes (Haizi) in Jiuzhaigou (animal and plant characteristics, water characteristics, water environment, and animal and plant environment) have a positive impact on restorativeness, with animal and plant characteristics exerting the most significant impact. In a comparison of the restorativeness between single-form natural visual elements, Ulrich et al. (39) found that water elements have more positive effects in terms of emotion regulation compared to vegetation elements. Low hydrodynamic landscapes (Haizi) in Jiuzhaigou are natural spaces containing rich flora and fauna and large areas of water. Plants and water can enhance mental restorativeness (40) as they can provide a comfortable environment by improving the microclimate, thus positively impacting the restorativeness of human health (41, 42). Our results revealed that for low hydrodynamic landscapes (Haizi) with abundant vegetation and large-area water elements, the restorative effects of animals and plants are greater than those of water bodies. In nature, water cannot exist independently from other features, such as plants and animals. Therefore, even spaces with water as the main landscape type have differences in waterscape vision due to distinct near- and distant-view backgrounds, including color composition (43). Plants and wildlife associated with water can strongly affect individuals' positive cognition of water spaces (44), with viewers feeling a sense of belonging and attachment (45, 46), thus enhancing the attractiveness of water landscapes (47). This is in line with the Biophilia Hypothesis, which holds that humans have an inherent tendency to get close to and love natural life (plants and animals) during the long-term evolution process. This tendency is related to survival instinct. Environments with a great diversity of plants and animals by water (e.g., trees, shrubs, flowers, butterflies, and birds) have a higher level of biodiversity, which can reduce mental stress and improve the environmental restorative effects (48). Jiuzhaigou, a World Natural Heritage Site, is renowned for its rich biodiversity and the presence of rare species (49). The low hydrodynamic landscapes (Haizi) in Jiuzhaigou boast abundant animal and plant resources, a diverse ecosystem, and a superior natural environment, all of which contribute significantly to the restoration of youth with high stress levels.

#### 4.4.2 Restorativeness of the characteristics of low hydrodynamic landscapes (Haizi)

The perceived depth and openness of water, as well as the density and level richness of plants, have positive effects on landscape restorativeness. In contrast, plant color richness is considered to have a negative effect on landscape restorativeness. Acquired knowledge and experience play an important role in people's understanding of the environment (50). When people can clearly perceive the depth of water, they can then determine the danger level of the water body and whether it is suitable for recreation (51). An open space is likely to provide a sense of security and control due to a wide view (52), which is in line with the need for viewing and sheltering (53). Environments with a wider view and less sheltering induce greater restorativeness than suburban park environments with a narrower view and more sheltering (54). Furthermore, the more visible trees, the greater the restorativeness (40). The density of green plants and the richness of plant layers are both related to the number of plants. Both high-density vegetation and richly layered plant communities can attract attention and evoke positive emotions (55). Our study finds that the higher the perceived plant color richness in space, the lower its perceptual restorative effects. This may be because lower plant color richness makes it easier to create harmonious spatial colors that do not induce a sense of disorder (56). It also facilitates improved coordination of plants with the surrounding features, resulting in a more orderly environment that aids in reducing the need for directed attention and promotes relaxation in both physiological and psychological states. Consequently, plant color serves as a crucial indicator in enhancing public mental health (57).

#### 4.4.3 Restorativeness of environmental elements in low hydrodynamic landscapes (Haizi)

As a component of water reflection, clouds have a significant impact on restorativeness. Reflection in water is the embodiment of symmetrical beauty and form, placing minimal strain on the eyes of viewers. The comfortable and labor-saving perceptual experience can bring viewers pleasure. Moreover, due to the balanced rhythm among symmetrical objects, viewers can get a sense of order as well as a feeling of control during the perceptual experience. The pursuit of beauty in form by humans is based on a sense of order and security, which is also the most fundamental aesthetic consciousness of humans (58). For waterscapes, watercolor is a significant factor affecting the perceptual properties of water. According to neuroaesthetics, the optic nerve responds fastest to color in external stimuli, with responses reported as 30 ms faster than those to motion (59). We identified green water bodies as having a greater perceptual restorativeness for youth. Blue and green are the universal color preferences among people due to genetic codes related to blue skies and green nature in human evolution. In particular, these two colors exert a sensory calming effect (60).

There is a negative correlation between plank roads over water and restorativeness. When people perceive the environment, they are more inclined to perceive the overall picture. However, plank roads over water destroy the integrity of the visual perception of landscapes for viewers at a distance. In addition, being exposed to remote viewing and becoming a member of the observed objects can reduce individuals' sense of security, which goes against humans' “viewing–sheltering” needs. Plank roads over water may also impact the microbial environment and living habits of aquatic animals due to the materials and technology used to construct the roads (e.g., unpleasant smells). This may lower the level of biodiversity and even damage the ecological environment, further affecting the restorativeness of water spaces. Therefore, it is necessary to carefully use the facilities of key ecological environments such as the Jiuzhaigou World Natural Heritage Site to protect the long-term ecosystem value.

The species richness of animals and plants is a key indicator of general species richness. Parasympathetic nerve activity is stronger in environments with higher species richness, which can significantly reduce the anxiety level of youth (61) and improve the restorative effects for introverts (62). Enhancing the number of plant and animal species can increase human preferences and induce other positive perceptions. The greater the number of plant and animal species, the higher the perceived naturalness (56, 63). Moreover, planting more vegetation in a space can increase its influence in reducing anxiety (64). In low hydrodynamic landscapes (Haizi), trees significantly increase people's preference for interacting with nature, effectively enhancing the restorativeness of the environment. Rich plant and animal species provide rich biodiversity and are associated with positive emotional effects, suggesting that higher biodiversity can lead to greater restorativeness and lower physiological stress (65).

## 5 Research summary and prospects

This study investigates the restorative effects of different hydrodynamic landscapes in Jiuzhaigou World Natural Heritage Site on youth in terms of audiovisual perception. According to the results, natural blue–green spaces with different hydrodynamic intensities are crucial to improving the health and well-being of the population. Moreover, hydrodynamic landscapes have an impact on the restorativeness of human physio-psychological health. In the same environments, different landscape elements of audiovisual perception have different restorative effects. This study identified the differences in the restorativeness of numerous hydrodynamic landscapes in Jiuzhaigou and determined the restorative components, characteristics, and elements of low hydrodynamic landscapes. Despite the progress made, this work is associated with several limitations. For example, the participants in this study were youth from China, yet it is necessary to take into consideration participants from a wider range of social, demographic, occupational, and cultural backgrounds, as well as those with different health conditions. In addition, the experimental material was collected in summer, with no data on the landscapes of the other three seasons. However, the landscapes in Jiuzhaigou vary with the season. Thus, further studies can explore whether the differences in season can induce variations in the psychological restorative effects of hydrodynamic landscapes on youth and other populations. Future work should also focus on the landscape optimization of blue–green spaces such as Jiuzhaigou and other World Natural Heritage Sites by fully accounting for the hydrodynamic intensities of waterscapes, audiovisual interaction stimuli, and other aspects to enrich people's perceived experience. Furthermore, landscape elements can be improved through targeted optimization to create landscape spaces that enhance individuals' restorative potential, which is conducive to creating a high-quality environment for the tourism industry. It is critical to determine effective measures to protect the natural environment of Jiuzhaigou and to define standards for new constructions that maintain an ecological balance. Pro-environmental behavior should also be advocated among the population, and cognitive attitudes and behavior toward protecting the environment should be encouraged and deepened for individuals.

## Data Availability

The original contributions presented in the study are included in the article/Supplementary Material, further inquiries can be directed to the corresponding authors.

## Appendix A

**Table:**

Dimensions of perceptual restorativeness	Question item	Agreement level				
		1	2	3	4	**5**
Remoteness	This place makes me feel vulgar					
	This place provides me space to break from my everyday routine and allows me to rest					
	I can fully relax here					
	This place helps me relax my tense mood					
	This place makes me feel free from the constraints of work and daily life					
Extensibility	The surrounding scenes are harmonious					
	I am curious about the unseen landscapes in the scenery here					
	Here my imagination can be stretched					
	The elements of the landscapes here are similar					
Charmingness	The place is attractive					
	I can visually explore and discover more here					
	The visual scenes here are charming					
	I would like to spend more time watching the scenes here					
Compatibility	I can engage in the activities I like here					
	I can quickly adapt to the scenes here					
	This place brings me a sense of belonging					
	I can find ways to enjoy myself here					
	What I want to do here is consistent with the environment					

## Appendix B

**Table:**

Research object	Overall perceptual restorativeness	Characteristics of low hydrodynamic landscapes (Haizi)			
		Component	Perceptual restorativeness	Characteristic	Perceptual restorativeness
Low hydrodynamic landscapes (Haizi)	1 2 3 4 5 6 7	Water characteristics	1 2 3 4 5 6 7	Water transparency (underwater visibility) (high/low)	1 2 3 4 5 6 7
				Perceived depth of water (deep/shallow)	1 2 3 4 5 6 7
				Proximity to people (high/low)	1 2 3 4 5 6 7
				Degree of openness (high/low)	1 2 3 4 5 6 7
				Water surface complexity (high/low)	1 2 3 4 5 6 7
				Color richness (high/low)	1 2 3 4 5 6 7
		Plant and animal characteristics	1 2 3 4 5 6 7	Plant density (high/low)	1 2 3 4 5 6 7
				Plant level richness (high/low)	1 2 3 4 5 6 7
				Plant color richness (high/low)	1 2 3 4 5 6 7
				Plant morphological richness (high/low)	1 2 3 4 5 6 7
				Animal species richness (high/low)	1 2 3 4 5 6 7

## Appendix C

**Table:**

Research object	Overall perceptual restorativeness	Environment of Haizi					
		Component	Perceptual restorativeness	Element	Perceptual restorative-ness	Composition	Perceptual restorative-ness
Low hydrodynamic landscapes (Haizi)	1 2 3 4 5 6 7	Water environment	1 2 3 4 5 6 7	Water color	1 2 3 4 5 6 7	Blue body	1 2 3 4 5 6 7
						Green body	1 2 3 4 5 6 7
						Yellow body	1 2 3 4 5 6 7
				Waterfront elements	1 2 3 4 5 6 7	Mountain stones	1 2 3 4 5 6 7
						Grassy slopes	1 2 3 4 5 6 7
						Dense Forest	1 2 3 4 5 6 7
						Trees	1 2 3 4 5 6 7
						Wooden plank road	1 2 3 4 5 6 7
				Water shape	1 2 3 4 5 6 7	Strip	1 2 3 4 5 6 7
						Rectangle	1 2 3 4 5 6 7
						Oval	1 2 3 4 5 6 7
						Peacock shape	1 2 3 4 5 6 7
						Irregular shape	1 2 3 4 5 6 7
						Bead net shape	1 2 3 4 5 6 7
				Reflection	1 2 3 4 5 6 7	Cloud	1 2 3 4 5 6 7
						Plants on the waterfront	1 2 3 4 5 6 7
						Plants in water	1 2 3 4 5 6 7
						Mountain	1 2 3 4 5 6 7
				Individual-form scene	1 2 3 4 5 6 7	Plank roads on the waterfront	1 2 3 4 5 6 7
						Plank roads over water	1 2 3 4 5 6 7
						Viewing platform (with guardrail)	1 2 3 4 5 6 7
						Fallen tree	1 2 3 4 5 6 7
		Plant and animal environment	1 2 3 4 5 6 7	Plant species	1 2 3 4 5 6 7	Aquatic plant	1 2 3 4 5 6 7
						Submerged plant	1 2 3 4 5 6 7
						Humidogene shrub	1 2 3 4 5 6 7
						Humidogene tree	1 2 3 4 5 6 7
				Animal species	1 2 3 4 5 6 7	Terrestrial	1 2 3 4 5 6 7
						Fish	1 2 3 4 5 6 7

**Appendix Table 7:** Multiple linear regression analysis of the restorativeness of water characteristics.

Model	Non-standard coefficient		Standard coefficient	*T*-value	*P*-value	95.0% Confidence interval for beta		Collinear statistics		*R*²	Adjusted *R*²
	B	Standard error	Beta			Minimum value	Maximum value	Tolerance	VIF		
(Constant)	1.978	.785		2.520	.018*	.365	3.591			.489	.390
Clearness	.046	.114	.066	.402	.691	−.189	.281	.736	1.358		
Perceived depth	.240	.104	.377	2.303	.030*	.026	.454	.734	1.362		
Openness	.178	.105	.281	1.699	.101	−.037	.393	.721	1.387		
Surface complexity	.084	.102	.130	.821	.419	−.126	.294	.780	1.282		
Color richness	.144	.119	.207	1.213	.236	−.100	.388	.675	1.481		
Implicit variable: restorativeness of the water characteristics											

**P* < 0.05, ***P* < 0.01, ****P* < 0.001.

**Appendix Table 8:** Multiple linear regression analysis of the restorativeness of the plant and animal characteristics.

Model	Non-standard coefficient		Standard coefficient	*T*-value	*P*-value	95.0% Confidence interval for beta		Collinear statistics		*R*²	Adjusted *R*²
	B	Standard error	Beta			Minimum value	Maximum value	Tolerance	VIF		
(Constant)	.934	1.026		.910	.371	−1.172	3.039			.462	.382
Plant density	.419	.159	.477	2.635	.014*	.093	.745	.608	1.644		
Plant level richness	.416	.181	.376	2.293	.030*	.044	.788	.740	1.352		
Plant color richness	−.163	.193	−.178	−.842	.407	−.559	.234	.446	2.242		
Plant form richness	.091	.181	.096	.503	.619	−.281	.464	.545	1.836		
Implicit variable: restorativeness of the plant and animal characteristics											

**P* < 0.05, ***P* < 0.01, ****P* < 0.001.

**Appendix Table 9:** Multiple linear regression analysis of the restorativeness of water environments.

Model	Non-standard coefficient		Standard coefficient	*T*-value	*P*-value	95.0% Confidence interval for beta		Collinear statistics		*R*²	Adjusted *R*²
	B	Standard error	Beta			Minimum value	Maximum value	Tolerance	VIF		
(Constant)	.739	.893		.828	.416	−1.105	2.583			.641	.566
Water color	.348	.185	.300	1.884	.072	−.033	.729	.591	1.691		
Waterfront elements	.347	.145	.436	2.387	.025*	.047	.647	.448	2.231		
Water shape	.052	.120	.063	.432	.670	−.195	.299	.702	1.425		
Water reflection	.373	.121	.517	3.083	.005**	.123	.622	.532	1.881		
Individual scene	−.219	.120	−.330	−1.827	.080	−.466	.028	.460	2.174		
Implicit variable: restorativeness of water environments											

**P* < 0.05, ***P* < 0.01, ****P* < 0.001.

**Appendix Table 10:** Multiple linear regression analysis of the restorativeness of the water reflection components.

Model	Non-standard coefficient		Standard coefficient	*T*	*P*	95.0% Confidence interval for beta		Collinear statistics		*R*²	Adjusted *R*²
	B	Standard error	Beta			Minimum value	Maximum value	Tolerance	VIF		
(Constant)	1.101	.885		1.244	.224	−.715	2.917			.585	.523
Plants on the water	−.023	.141	−.028	−.161	.873	−.313	.267	.496	2.015		
Waterfront plants	.219	.159	.258	1.377	.180	−.107	.545	.439	2.280		
Clouds	.435	.209	.438	2.083	.047*	.007	.864	.348	2.870		
Mountains	.201	.170	.233	1.178	.249	−.149	.550	.393	2.542		
Implicit variable: restorativeness of water reflection components											

**P* < 0.05, ***P* < 0.01, ****P* < 0.001.

**Appendix Table 11:** Multiple linear regression analysis of the restorativeness of the watercolor components.

Model	Non-standard coefficient		Standard coefficient	T-value	*P*-value	95.0% Confidence interval for beta		Collinear statistics		*R*²	Adjusted *R*²
	B	Standard error	Beta			Minimum value	Maximum value	Tolerance	VIF		
(Constant)	2.238	.698		3.208	.003**	.809	3.667			.487	.432
Mainly blue	.222	.091	.341	2.451	.021*	.036	.407	.948	1.055		
Mainly green	.306	.091	.473	3.379	.002**	.121	.492	.935	1.070		
Mainly yellow	.154	.064	.332	2.409	.023*	.023	.285	.966	1.035		
Implicit variable: restorativeness of water color components											

**P* < 0.05, ***P* < 0.01, ****P* < 0.001.

**Appendix Table 12:** Multiple linear regression analysis of the restorativeness of the individual scene components. Values indicate negative impacts of the individual scene components, and the higher the value, the greater the negative impact.

Model	Non-standard coefficient		Standard coefficient	T-value	*P*-value	95.0% Confidence interval for beta		Collinear statistics		*R*²	Adjusted *R*²
	B	Standard error	Beta			Minimum value	Maximum value	Tolerance	VIF		
(Constant)	1.369	1.021		1.341	.191	−.726	3.464			.396	.306
Plank roads by the shore	.236	.163	.239	1.452	.158	−.098	.570	.828	1.208		
Plank roads over water	.381	.172	.420	2.212	.036*	.028	.735	.622	1.608		
Viewing platform with guardrails	.065	.149	.076	.440	.663	−.240	.371	.743	1.345		
Fallen trees in the water	.051	.113	.073	.457	.652	−.180	.282	.883	1.132		
Implicit variable: restorativeness of individual scene components											

**P* < 0.05, ***P* < 0.01, ****P* < 0.001.

**Appendix Table 13:** Multiple linear regression analysis of the restorativeness of plant and animal environments.

Model	Non-standard coefficient		Standard coefficient	T-value	*P*-value	95.0% Confidence interval for beta		Collinear statistics		*R*²	Adjusted *R*²
	B	Standard error	Beta			Minimum value	Maximum value	Tolerance	VIF		
(Constant)	−.674	.556		−1.213	.236	−1.815	.467			.828	.815
Plant species	.543	.085	.569	6.410	.000***	.369	.716	.810	1.235		
Animal species	.547	.096	.504	5.680	.000***	.349	.744	.810	1.235		
Implicit variable: restorativeness of plant and animal environments											

**P* < 0.05, ***P* < 0.01, ****P* < 0.001.

**Appendix Table 14:** Multiple linear regression analysis of the restorativeness of plant species.

Model	Non-standard coefficient		Standard coefficient	T-value	*P*-value	95.0% Confidence interval for beta		Collinear statistics		*R*²	Adjusted *R*²
	B	Standard error	Beta			Minimum value	Maximum value	Tolerance	VIF		
(Constant)	.435	.719		.605	.550	−1.041	1.911			.711	.668
Submerged plants	.044	.090	.062	.483	.633	−.142	.229	.642	1.558		
Emergent plants	.212	.083	.305	2.546	.017*	.041	.382	.749	1.335		
Wetland shrubs	.373	.135	.415	2.753	.010**	.095	.651	.472	2.119		
Wetland trees	.352	.138	.328	2.545	.017*	.068	.636	.644	1.554		
Implicit variable: restorativeness of plant species											

**P* < 0.05, ***P* < 0.01, ****P* < 0.001.

**Appendix Table 15:** Multiple linear regression analysis of the restorativeness of animal species.

Model	Non-standard coefficient		Standard coefficient	T-value	*P*-value	95.0% Confidence interval for beta		Collinear statistics		*R*²	Adjusted *R*²
	B	Standard error	Beta			Minimum value	Maximum value	Tolerance	VIF		
(Constant)	.760	.864		.880	.386	−1.007	2.526			.540	.509
Terrestrial	.455	.113	.509	4.040	.000***	.225	.686	.999	1.001		
Fish	.433	.106	.516	4.094	.000***	.216	.649	.999	1.001		
Implicit variable: restorativeness of animal species											

**P* < 0.05, ***P* < 0.01, ****P* < 0.001.
