# Supplementary material for: Exploring the restorativeness of different hydrodynamic landscapes in world natural heritage sites
Source: Front Child Adolesc Psychiatry. 2025 Feb 12;4:1506392. doi: 10.3389/frcha.2025.1506392 (PMC11860883; doi:10.3389/frcha.2025.1506392)
Supplement: Supplementary file 1 [file Table1.docx]

**Appendix A**

| **Dimensions of perceptual restorativeness** | **Question item** | **Agreement level** | | | | |
| --- | --- | --- | --- | --- | --- | --- |
|  |  | 1 | 2 | 3 | 4 | 5 |
| **Remoteness** | This place makes me feel vulgar. |  |  |  |  |  |
|  | This place provides me space to break from my everyday routine and allows me to rest. |  |  |  |  |  |
|  | I can fully relax here. |  |  |  |  |  |
|  | This place helps me relax my tense mood. |  |  |  |  |  |
|  | This place makes me feel free from the constraints of work and daily life. |  |  |  |  |  |
| **Extensibility** | The surrounding scenes are harmonious. |  |  |  |  |  |
|  | I am curious about the unseen landscapes in the scenery here. |  |  |  |  |  |
|  | Here my imagination can be stretched. |  |  |  |  |  |
|  | The elements of the landscapes here are similar. |  |  |  |  |  |
| **Charmingness** | The place is attractive. |  |  |  |  |  |
|  | I can visually explore and discover more here. |  |  |  |  |  |
|  | The visual scenes here are charming. |  |  |  |  |  |
|  | I would like to spend more time watching the scenes here. |  |  |  |  |  |
| **Compatibility** | I can engage in the activities I like here. |  |  |  |  |  |
|  | I can quickly adapt to the scenes here. |  |  |  |  |  |
|  | This place brings me a sense of belonging. |  |  |  |  |  |
|  | I can find ways to enjoy myself here. |  |  |  |  |  |
|  | What I want to do here is consistent with the environment. |  |  |  |  |  |

**Appendix B**

| Research object | Overall perceptual restorativeness | Characteristics of low hydrodynamic landscapes (Haizi) | | | |
| --- | --- | --- | --- | --- | --- |
|  |  | Component | Perceptual restorativeness | Characteristic | Perceptual restorativeness |
| Low hydrodynamic landscapes (Haizi) | 1 2 3 4 5 6 7 | Water characteristics | 1 2 3 4 5 6 7 | Water transparency (underwater visibility) (high/low) | 1 2 3 4 5 6 7 |
|  |  |  |  | Perceived depth of water (deep/shallow) | 1 2 3 4 5 6 7 |
|  |  |  |  | Proximity to people (high/low) | 1 2 3 4 5 6 7 |
|  |  |  |  |  |  |
|  |  |  |  | Degree of openness (high/low) | 1 2 3 4 5 6 7 |
|  |  |  |  |  |  |
|  |  |  |  | Water surface complexity (high/low) | 1 2 3 4 5 6 7 |
|  |  |  |  |  |  |
|  |  |  |  | Color richness (high/low) | 1 2 3 4 5 6 7 |
|  |  |  |  |  |  |
|  |  | Plant and animal characteristics | 1 2 3 4 5 6 7 | Plant density (high/low) | 1 2 3 4 5 6 7 |
|  |  |  |  |  |  |
|  |  |  |  | Plant level richness (high/low) | 1 2 3 4 5 6 7 |
|  |  |  |  |  |  |
|  |  |  |  | Plant color richness (high/low) | 1 2 3 4 5 6 7 |
|  |  |  |  |  |  |
|  |  |  |  | Plant morphological richness (high/low) | 1 2 3 4 5 6 7 |
|  |  |  |  |  |  |
|  |  |  |  | Animal species richness (high/low) | 1 2 3 4 5 6 7 |
|  |  |  |  |  |  |

**Appendix C**

| Research object | Overall perceptual restorativeness | Environment of Haizi | | | | | |
| --- | --- | --- | --- | --- | --- | --- | --- |
|  |  | Component | Perceptual restorativeness | Element | Perceptual restorative-ness | Composition | Perceptual restorative-ness |
| Low hydrodynamic landscapes (Haizi) | 1 2 3 4 5 6 7 | Water environment | 1 2 3 4 5 6 7 | Water color | 1 2 3 4 5 6 7 | Blue body | 1 2 3 4 5 6 7 |
|  |  |  |  |  |  | Green body | 1 2 3 4 5 6 7 |
|  |  |  |  |  |  | Yellow body | 1 2 3 4 5 6 7 |
|  |  |  |  | Waterfront elements | 1 2 3 4 5 6 7 | Mountain stones | 1 2 3 4 5 6 7 |
|  |  |  |  |  |  | Grassy slopes | 1 2 3 4 5 6 7 |
|  |  |  |  |  |  | Dense Forest | 1 2 3 4 5 6 7 |
|  |  |  |  |  |  | Trees | 1 2 3 4 5 6 7 |
|  |  |  |  |  |  | Wooden plank road | 1 2 3 4 5 6 7 |
|  |  |  |  | Water shape | 1 2 3 4 5 6 7 | Strip | 1 2 3 4 5 6 7 |
|  |  |  |  |  |  | Rectangle | 1 2 3 4 5 6 7 |
|  |  |  |  |  |  | Oval | 1 2 3 4 5 6 7 |
|  |  |  |  |  |  | Peacock shape | 1 2 3 4 5 6 7 |
|  |  |  |  |  |  | Irregular shape | 1 2 3 4 5 6 7 |
|  |  |  |  |  |  | Bead net shape | 1 2 3 4 5 6 7 |
|  |  |  |  | Reflection | 1 2 3 4 5 6 7 | Cloud | 1 2 3 4 5 6 7 |
|  |  |  |  |  |  | Plants on the waterfront | 1 2 3 4 5 6 7 |
|  |  |  |  |  |  | Plants in water | 1 2 3 4 5 6 7 |
|  |  |  |  |  |  | Mountain | 1 2 3 4 5 6 7 |
|  |  |  |  | Individual-form scene | 1 2 3 4 5 6 7 | Plank roads on the waterfront | 1 2 3 4 5 6 7 |
|  |  |  |  |  |  | Plank roads over water | 1 2 3 4 5 6 7 |
|  |  |  |  |  |  | Viewing platform （with guardrail） | 1 2 3 4 5 6 7 |
|  |  |  |  |  |  | Fallen tree | 1 2 3 4 5 6 7 |
|  |  | Plant and animal environment | 1 2 3 4 5 6 7 | Plant species | 1 2 3 4 5 6 7 | Aquatic plant | 1 2 3 4 5 6 7 |
|  |  |  |  |  |  | Submerged plant | 1 2 3 4 5 6 7 |
|  |  |  |  |  |  | Humidogene shrub | 1 2 3 4 5 6 7 |
|  |  |  |  |  |  | Humidogene tree | 1 2 3 4 5 6 7 |
|  |  |  |  | Animal species | 1 2 3 4 5 6 7 | Terrestrial | 1 2 3 4 5 6 7 |
|  |  |  |  |  |  | Fish | 1 2 3 4 5 6 7 |
