# Supplementary material for: Exploring the restorativeness of different hydrodynamic landscapes in world natural heritage sites
Source: Front Child Adolesc Psychiatry. 2025 Feb 12;4:1506392. doi: 10.3389/frcha.2025.1506392 (PMC11860883; doi:10.3389/frcha.2025.1506392)
Supplement: Supplementary file 2 [file Table2.docx]

1. Audiovisully perceived physio-restorativeness is positively related to hydrodynamic energy.
2. Sounds of water and birds can add physio-restorativeness to high-energy hydrodynamic landscapes.
3. Form-rich combined space of medium hydrodynamic energy has the greatest psycho-restorativeness.
4. Rich visual stimuli can add restorativeness to landscapes with low and medium hydrodynamic energy.
5. Plant and animal resources feature greater restorativeness than water resource in waterscapes.
