# Supplementary material for: Exploring the restorativeness of different hydrodynamic landscapes in world natural heritage sites
Source: Front Child Adolesc Psychiatry. 2025 Feb 12;4:1506392. doi: 10.3389/frcha.2025.1506392 (PMC11860883; doi:10.3389/frcha.2025.1506392)
Supplement: Supplementary file 3 [file Table3.docx]

**Tittle Page**

**Tittle:** **Exploring the Restorativeness of Different Hydrodynamic Landscapes in World Natural Heritage Sites**

Ping Zhang ^a#^, Tongyao Zhang ^a#^, Zexuan Chen ^b#^, Qianyi He ^a^, Ke Luo ^a^ , Jinpeng Li ^a^, Yanbin Yang ^a^, Qingjie Zhang ^a^, Xuan Wang ^a^, Limin Han ^c^, Mingze Chen ^d^, Fuping Zhao ^a^, Xiaoqing He ^a^, Saixin Cao ^a^, Xiaoqing Xu ^e^, Guangyu Wang ^d^ * and Xi Li ^a^ *

^a^ College of Landscape Architecture, Sichuan Agricultural University, Chengdu ,611130, China;

^b^ School of Architecture and Urban Planning, Chongqing University, Chongqing, 400030, China;

^c^ School of International Education, Chengdu Agricultural College, Chengdu, 610101, China;

^d^ Faculty of Forestry, Department of Forest and Resources Management, University of British Columbia, 2424 Main Mall, Vancouver, BC V6T 1Z4, Canada.

^e^ Department of Landscape Architecture, School of Architecture and Urban Planning, Tongji University, Shanghai, 200092, China;

* Correspondence: [guangyu.wang@ubc.ca;](mailto:guangyu.wang@ubc.ca;) [lixi@sicau.edu.cn](mailto:lixi@sicau.edu.cn)

^#^ These authors contributed equally to this work.

*Corresponding Author:

**Guangyu, Wang**

The University of British Columbia

-Vancouver Campus, Forest Resources Management,Vancouver, V6T 1Z4,CA

E-mail address: guangyu.wang@ubc.ca

**Xi Li**

College of Landscape Architecture, Sichuan Agricultural University, Chengdu, 611130, China

1. mail address: [lixi@sicau.edu.cn](mailto:lixi@sicau.edu.cn)

**CRediT authorship contribution statement**

Methodology, Q.H., Z.C., Q.Z, T.Z., and P.Z.; data curation, K.L, S.C. and X.W.; formal analysis, J.L., Y.Y., M.C. and G.W.; funding acquisition, G.W.,X.L. and X.X.; investigation Z.C., Q.H., K.L, X.W., T.Z. and P.Z.; project administration P.Z., T.Z. and Q.H.; resources, P.Z., T.Z., X.X., F.Z., X.H. and X.L.; supervision, G.W., X.L.; writing original draft, Z.C., Q.H., T.Z., and P.Z.; writing reviewing & editing, Q.H., Z.C., L.H. and Q.Z.. All authors have read and agreed to the published version of the manuscript.
