# Supplementary material for: Exploring the restorativeness of different hydrodynamic landscapes in world natural heritage sites
Source: Front Child Adolesc Psychiatry. 2025 Feb 12;4:1506392. doi: 10.3389/frcha.2025.1506392 (PMC11860883; doi:10.3389/frcha.2025.1506392)
Supplement: Supplementary file 4 [file Table4.docx]

**Declaration of Interest Statement**

The authors declare that they have no known competing financial interests or personal relationships that could have appeared to influence the work reported in this paper.
