# Supplementary material for: Exploring the restorativeness of different hydrodynamic landscapes in world natural heritage sites
Source: Front Child Adolesc Psychiatry. 2025 Feb 12;4:1506392. doi: 10.3389/frcha.2025.1506392 (PMC11860883; doi:10.3389/frcha.2025.1506392)
Supplement: Supplementary file 5 [file Table5.docx]

**Acknowledgments:**

This work was supported by Research on the constructing of multi-sensory perception system and its health effects in Jiuzhaigou World Heritage Site under the background of post-epidemic situation（22GJHZ0142）
