# Supplementary material for: Exploring the restorativeness of different hydrodynamic landscapes in world natural heritage sites
Source: Front Child Adolesc Psychiatry. 2025 Feb 12;4:1506392. doi: 10.3389/frcha.2025.1506392 (PMC11860883; doi:10.3389/frcha.2025.1506392)
Supplement: Supplementary file 6 [file Table6.docx]

**Informed Consent Statement:**

Informed consent was obtained from all subjects involved in the study.
