# Supplementary material for: Exploring the restorativeness of different hydrodynamic landscapes in world natural heritage sites
Source: Front Child Adolesc Psychiatry. 2025 Feb 12;4:1506392. doi: 10.3389/frcha.2025.1506392 (PMC11860883; doi:10.3389/frcha.2025.1506392)
Supplement: Supplementary file 7 [file Table7.docx]

**Institutional Review Board Statement:**

The study was conducted in accordance with the Declaration of Helsinki and was approved by the Science and Technology Management Office of Sichuan Agricultural University (Oct. 2,2023).
